# Fibulin 1 is downregulated through promoter hypermethylation in gastric cancer

**DOI:** 10.1038/sj.bjc.6604760

**Published:** 2008-11-04

**Authors:** Y Y Cheng, H Jin, X Liu, J M T Siu, Y P Wong, E K O Ng, J Yu, W-k Leung, J J Y Sung, F K L Chan

**Affiliations:** 1 Department of Medicine and Therapeutics, Institute of Digestive Disease, Faculty of Medicine, Li Ka Shing Institute of Health Sciences, The Chinese University of Hong Kong, Hong Kong SAR, China

**Keywords:** *FBLN1*, methylation, tumour suppressor gene, gastric cancer

## Abstract

Tumour suppressor genes (TSGs) were frequently inactivated through promoter hypermethylation in gastric carcinoma as well as pre-malignant gastric lesions, suggesting that promoter hypermethylation can be used as a marker to define novel TSGs and also biomarkers for early detection of gastric cancer. In an effort to search for such genes aberrantly methylated in gastric cancer development, fibulin 1 (FBLN1) was found as a candidate TSG epigenetically downregulated in gastric cancer. FBLN1 expression was downregulated in all of gastric cancer cell lines used (100%, 7 out of 7) and the primary gastric carcinoma tissues (84%, 86 out of 102) and significantly restored after pharmacological demethylation. Hypermethylation of the FBLN1 promoter was frequently (71%, 5 out of 7) detected in gastric cancer cell lines and primary gastric carcinoma tissues. Ectopic expression of FBLN1 led to the growth inhibition of gastric cancer cells through the induction of apoptosis. In summary, FBLN1 was identified as a novel candidate TSG epigenetically downregulated in gastric cancer.

Gastric cancer is the second most important cause of cancer death worldwide. Pathogenesis of gastric adenocarcinoma is believed to be a stepwise process resulting from aberrant oncogene activation and tumour suppressor gene (TSG) inactivations (Hanahan and Weinberg, 2000; Ponder, 2001; Ushijima and Sasako, 2004). Oncogenes, once activated, can promote carcinogenesis by conferring growth advantages. In contrast, TSGs have tumour-inhibitory functions and need to be inactivated during carcinogenesis. Oncogenes are activated mainly through genetic alterations, whereas aberrant TSG inactivation can result from both genetic and epigenetic alterations. Epigenetic alterations such as promoter hypermethylation can lead to the transcriptional silencing of TSGs, which is important for preventing cancer development. Given that most, if not all, TSGs can be inactivated through promoter hypermethylation in human cancers, promoter hypermethylation has been recognised as one of the most important markers for identifying novel TSGs (Jones and Baylin, 2002; Esteller, 2008). Moreover, promoter hypermethylation of tumour-related genes has also been proposed as a novel biomarker for detecting cancer and predicting prognosis (Jones and Baylin, 2007). We and others have shown earlier that many tumour-related genes were frequently silenced through promoter hypermethylation in gastric carcinoma as well as pre-malignant gastric lesions (Leung *et al*, 2001; Lee *et al*, 2002; To *et al*, 2002; Chan *et al*, 2004; Choi and Wu, 2005; Chan and Rashid, 2006; Sato and Meltzer, 2006; Cheng *et al*, 2007; Ushijima, 2007; Vogiatzi *et al*, 2007), suggesting that promoter hypermethylation can be utilised as biomarkers for early detection of gastric cancer.

In an attempt to identify novel TSGs silenced through promoter hypermethylation in gastric carcinogenesis and novel epigenetic biomarkers for the detection of gastric cancer, we have profiled genes epigenetically silenced in a panel of gastric cancer cell lines. We indeed found that many TSGs well known to be epigenetically silenced in gastric cancer, such as SFRP2 (secreted frizzled-related protein 2), PCDH10 (protocadherin 10), DKK3 (dickkopf homologue 3) and UCHL1 (ubiquitin carboxyl-terminal esterase L1), were markedly upregulated after pharmacological demethylation in many gastric cancer cell lines. In this study, we focused on fibulin 1 (FBLN1), which was significantly upregulated after pharmacological demethylation in all (5 out of 5) gastric cancer cell lines used.

Fibulin 1 belongs to a growing family of extracellular glycoproteins with distinctive features of a fibulin-type C-terminal domain preceded by tandem epidermal growth factor-like modules (Kobayashi *et al*, 2007). Fibulins have been shown to modulate cell morphology, growth, adhesion and motility. Dysregulation of certain FBLNs occurs in a range of human cancers, such as prostate cancer (Gallagher *et al*, 2005). Among them, FBLN1 and FBLN2 (fibulin 2) were found to be downregulated and acted as tumour suppressive genes in certain cancers such as prostate cancer and breast cancer (Wlazlinski *et al*, 2007; Yi *et al*, 2007). However, whether FBLN1 functions as a TSG in gastric cancer remains unknown.

## Materials and methods

### Cell lines, primary tissues and RNA/DNA extraction

Seven gastric cancer cell lines (AGS, Kato III, MKN28, MKN45, SNU1, SNU16 and NCI-N87) were obtained from Riken Gene Bank (Tsukuba, Ibaraki, Japan) and American Type Culture Collection (ATCC, Manassas, VA, USA). Unless specifically indicated, cells were cultured in RPMI-1640 medium (Invitrogen, Carlsbad, CA, USA) supplemented with 10% foetal bovine serum at 5% CO_2_, 37°C and 95% humidity. For pharmacological demethylation, cells were treated with 5 *μ*M 5-aza-2′-deoxycytidine (Aza) (Sigma, St Louis, MO, USA) for 3 consecutive days. 5-Aza-2′-deoxycytidine was replenished every 24 h. An equivalent concentration of the vehicle (DMSO) was used as the control. All primary tissues, including 102 gastric adenocarcinomas and 10 normal gastric specimens, were obtained from the Endoscopy Centre of the Prince of Wales Hospital, The Chinese University of Hong Kong. All specimens were immediately snap-frozen in liquid nitrogen and stored at −80°C until further processing. All subjects gave informed consent for obtaining the study materials. The study protocol was approved by the Clinical Research Ethics Committee of the Chinese University of Hong Kong.

Total RNA and genomic DNA were extracted using Trizol reagent (Invitrogen) according to the manufacturer's instructions.

### RT–PCR and quantitative real-time RT–PCR

Reverse transcription reaction was performed using 1 *μ*g of total RNA with Reverse Transcription System (Promega, Madison, WI, USA). The mRNA expression levels of the FBLN1 were determined by conventional RT–PCR with GoTaq polymerase (Promega) and quantitative real-time PCR using the SYBR Green Master Mix Kit and the ABI 7500 Real-Time PCR System (Applied Biosystems, Foster City, CA, USA). Glyceraldehyde-3-phosphate dehydrogenase was used as an internal control of RNA integrity. Primers used for FBLN1 RT–PCR were FBLN1-F: 5′-TGCGAATGCAAGACGG and FBLN1-R: 5′-CGTAGACGTTGGCACA.

### Bisulphite treatment of DNA, methylation-specific PCR

Methylation status of FBLN1 was determined by methylation-specific PCR (MSP) using bisulphate-modified genomic DNA as the template. Genomic DNA was bisulphite-treated with the Zymo DNA Modification Kit (Zymo Research, Orange, CA, USA) according to the protocol provided. Methylation-specific PCR was carried out for 40 cycles with annealing temperature at 62°C, as described earlier (Cheng *et al*, 2007). Methylation-specific primers were FBLN1M-F: 5′-attaggagattcgcggtttc and FBLN1M-R: 5′-gctccataaacgacgaacg, and the unmethylation-specific primers were FBLN1U-F: 5′-gattaggagatttgtggttttg and FBLN1U-R: 5′-cacactccataaacaacaaaca. All primers were confirmed earlier for not amplifying any non-bisulphite treated DNA.

### Construction of FBLN1 expression plasmids

The FBLN1 expression plasmid was constructed by cloning of the full-length FBLN1 open reading frame into mammalian expression vector pcDNA3.1. The full-length FBLN1 open reading frame was amplified from normal stomach cDNA using high-fidelity PFU DNA polymerase (Invitrogen). The sequence and orientation of the insert were confirmed by DNA sequencing.

### Western blot analysis

A total of 20 *μ*g of protein of each sample was resolved in 10% sodium dodecyl sulphate/polyacrylamide gel electrophoresis. The proteins on the polyacrylamide gel were transferred onto a nitrocellulose membrane (Amersham, Piscataway, NJ, USA). Polyclonal FBLN1 antibody (1 : 1000, 4°C, overnight; Santa Cruz Biotechnology, Santa Cruz, CA, USA) and the horseradish peroxidase-conjugated goat anti-rabbit secondary antibody (1 : 2000, room temperature, 1 h; Santa Cruz Biotechnology) were applied for the detection of FBLN1 proteins. The blot was visualised by using the enhanced chemiluminescence detection system (Amersham) according to the manufacturer's protocol. Human *β*-actin was used as the control of protein integrity.

### Cell growth assay

Transiently transfected MKN45 cells with empty pcDNA3.1 or pcDNA3.1-FBLN1 vector were used for the monolayer colony-formation assay. Cells were cultured overnight in a 12-well plate (1.0 × 10^5^ per well) and transfected with pcDNA3.1 or the FBLN1-expressing vector using FuGENE 6 (Roche, Mannheim, Germany). Forty-eight hours later, the transfectants were re-plated in triplicate and cultured for 10–15 days in complete RPMI-1640 medium containing G418 (400 *μ*g ml^−1^). Surviving colonies were stained with Gentian Violet after methanol fixation and visible colonies (⩾50 cells) were counted. The experiments were repeated three times.

### Flow cytometric analysis

Cell apoptosis was determined by the detection of sub-G1 distribution with flow cytometric analysis. Briefly, cells transfected with pcDNA3.1 or pcDNA3.1-FBLN1 vector were harvested and washed twice with PBS. The cells were then fixed in ice-cold ethanol for at least 1 h. After washing out ethanol, the fixed cells were treated with 0.01% RNase (10 mg ml^−1^; Sigma) for 10 min at 37°C and then stained with 0.05% propidium iodide for 20 min at 4°C in the dark. The cell cycle distribution was determined using a FACScan flow cytometer (Becton Dickinson, Mountain View, CA, USA) and 10 000 cells were analysed with the MultiCycle software package (Phoenix, San Diego, CA, USA).

## Results

### Upregulation of FBLN1 expression in gastric cancer cell lines after Aza treatment

**Figure 1:**
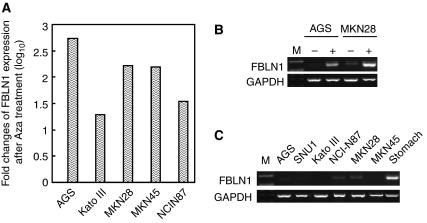
Pharmacological demethylation reactivates FBLN1 expression in gastric cancer cell lines. Relative FBLN1 expressions before and after Aza treatment were determined by quantitative real-time RT–PCR (**A**) and conventional RT–PCR (**B**). GAPDH was used to normalise the template amount. Representative results are shown. (**C**) Expression of FBLN1 in a panel of gastric cancer cell lines was determined by RT–PCR as in (**B**).

The expression of FBLN1 in gastric cancer cell lines, before and after Aza treatment, was determined by quantitative real-time RT–PCR. Fibulin 1 expression was significantly upregulated in all five gastric cancer cell lines (AGS, Kato III, MKN28, MKN45 and NCI-N87) after Aza treatment (Figure 1A and B). In addition, the expression of FBLN1 was downregulated in all five gastric cancer cell lines and two extra gastric cancer cell lines (SNU1 and SNU16) (Figure 1C), indicating that FBLN1 is most likely one novel TSG that is silenced through promoter hypermethylation in gastric cancer.

### Promoter hypermethylation of FBLN1 in gastric cancer cell lines

**Figure 2:**
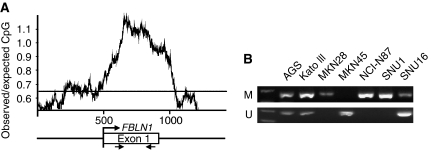
FBLN1 promoter is hypermethylated in gastric cancer cell lines. (**A**) Fibulin 1 has a typical CpG island around exon 1. CpG island was plotted by the GeneTool program. The position of MSP primers is indicated by arrows. (**B**) The methylation status of the FBLN1 promoter was determined by MSP. M: methylation; U: unmethylation.

A typical CpG island (CGI) was found around FBLN1 exon 1 using the following criteria: GC content >55%, ObsCpG/ExpCpG >0.65 and length >500 bp (Figure 2A). The methylation status of this CGI in gastric cancer cells was determined by MSP. As shown in Figure 2B, full methylation was detected in five gastric cancer cell lines, whereas the other two cell lines showed partial or no methylation of FBLN1 CGI.

### Downregulation and promoter hypermethylation of FBLN1 in primary tumours

**Figure 3:**
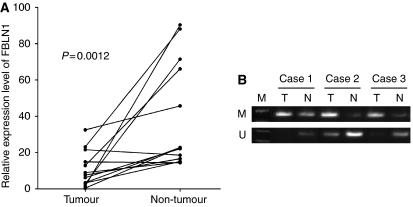
Fibulin 1 is downregulated and hypermethylated in primary gastric carcinoma tissues. (**A**) The expression of FBLN1 in gastric carcinoma and adjacent non-tumour tissues was determined by quantitative real-time RT–PCR as in Figure 1A. (**B**) The methylation status of the FBLN1 promoter in primary gastric carcinoma and adjacent non-tumour tissues was determined by MSP as in Figure 2B. Representative results are shown.

To further confirm the promoter CGI hypermethylation-mediated FBLN1 silencing in primary gastric carcinomas, FBLN1 expression and FBLN1 promoter methylation in primary gastric carcinoma tissues, adjacent non-tumour gastric tissues and normal gastric tissues were analysed by quantitative real-time RT–PCR and MSP, respectively. Fibulin 1 expression was significantly downregulated in most of the tumour tissues compared with adjacent non-tumour tissues (Figure 3A). In addition, promoter methylation was frequently detected in tumour samples but not in adjacent non-tumour and normal gastric tissue samples (Figure 3B), highlighting a tumour-specific hypermethylation of the FBLN1 promoter. There was no significant association between FBLN1 methylation and clinical characteristics such as age, gender, *Helicobacter pylori* infection, tumour grade, Lauren classification and differentiation (Table 1).

### Growth-inhibitory function of FBLN1

**Figure 4:**
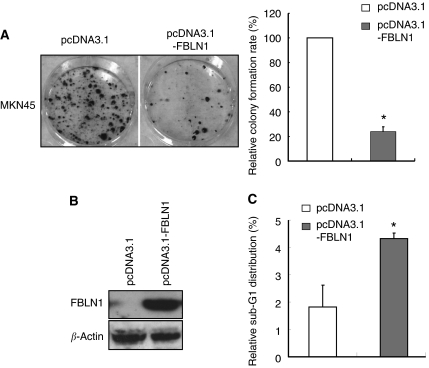
Fibulin 1 functions as a tumour suppressor gene in gastric cancer cell line MKN45. The effect of ectopic FBLN1 expression on tumour cell growth was investigated by the monolayer colony-formation assay (**A**). Quantitative analyses of colony numbers are shown in the right panel as values of mean±standard deviation. *P*-values were calculated using Student's *t*-test. The asterisk indicates statistically significant difference (*P*<0.01). The expression of FBLN1 after transfection was confirmed by western blotting analysis (**B**). (**C**) The rate of apoptosis before and after FBLN1 expression was determined by flow cytometry. Values are shown as mean±s.d. from triplicate experiments. *P*-values were calculated using Student's *t*-test. The asterisk indicates statistically significant difference (*P*<0.01).

Current data indicate that FBLN1 might have a tumour suppressive function. We investigated its tumour suppressive function by a gain-of-function strategy with the monolayer colony-formation assay. After re-expression of FBLN1 in MKN45 cells, the number of colonies formed on the plate was significantly reduced (*P*<0.01) (Figure 4A and B). The growth inhibition was caused by an increase in cell apoptosis, as the numbers of cells with sub-G1 distribution increased significantly after FBLN1 re-expression (Figure 4C).

## Discussion

Over the past several years, many TSGs have been found to be epigenetically inactivated in gastric cancer, indicating that epigenetic silencing of TSGs is one of the major molecular alterations in the process of gastric carcinogenesis (Lee *et al*, 2002; To *et al*, 2002; Choi and Wu, 2005; Chan and Rashid, 2006; Sato and Meltzer, 2006; Cheng *et al*, 2007). In this study, FBLN1 was identified as another candidate TSG whose inactivation may contribute to gastric carcinogenesis. It was frequently downregulated through promoter hypermethylation in gastric cancer cell lines and primary gastric carcinoma tissues. The ectopic expression of FBLN1 led to the growth inhibition of gastric cancer cells, indicating that FBLN1 functions as a novel TSG epigenetically silenced in gastric cancer.

There is growing evidence that hypermethylation of the TSG promoter represents one of the major molecular alterations in cancer development. Promoter hypermethylation can be used as a sensitive marker not only for TSG identification but also for cancer diagnosis and prognosis prediction (Jones and Baylin, 2002, 2007; Esteller, 2008). Fibulin 1 was identified in this study as a novel candidate TSG epigenetically inactivated in gastric cancer. However, we could not find any significant association of FBLN1 promoter methylation with clinical characteristics, including age, gender, *H. pylori* infection, tumour grade, Lauren classification and differentiation. This could be due to the limited resolution of methylation analysis used in this study. Other methylation analyses with higher resolution, such as quantitative methylation-specific analysis using sequenom or Taqman real-time PCR, are warranted to assess whether FBLN1 promoter methylation is useful for detecting early gastric cancer and predicting prognosis.

Although promoter methylation frequently inactivates FBLN1 in gastric cancer cell lines, we cannot exclude the presence of other mechanisms for the loss of function of FBLN1, such as defects in histone remodelling. For instance, the FBLN1 promoter was not significantly methylated in MKN45, which did not express FBLN1 (Figures 1C and 2B). In addition, we did not find any gastric cancer cell line with high level of FBLN1 expression, indicating that FBLN1 is inactivated mainly at the transcriptional level in gastric cancer. However, defects in FNLB1 maturation and mislocalisation have been found in other types of cancer, such as breast cancer (Pupa *et al*, 2004).

Interestingly, FBLN1 has four different variants that share a common promoter and a transcriptional start site (Pan *et al*, 1999). In this study, the longest isoform, which is the major isoform constitutively expressed in multiple tissues, was used for functional assays (Figure 4). The primers used in this study for RT–PCR analysis could reveal the expression status of all four isoforms, and no significant expression of FBLN1 was found in all gastric cancer cell lines (Figure 1C), indicating that the imbalanced expression of different isoforms is unlikely to be a relevant mechanism for FBLN1 inactivation in gastric cancer.

Current evidence suggests that FBLN1 plays a tumour suppressive role not only in gastric cancer but also in other cancers such as breast cancer and prostate cancer (Pupa *et al*, 2004; Wlazlinski *et al*, 2007). Fibulin 1 overexpression is correlated with better prognosis in breast cancer, and FBLN1 is downregulated in prostate cancer cells. The molecular mechanism of how FBLN1 suppresses tumorigenesis is not yet fully understood. Its expression can suppress cell motility and inhibit the phosphorylation of extracellular signal-regulated kinase and myosin light chain (Twal *et al*, 2001). Moreover, FBLN1 can reduce the intracellular calcium level, which is important for the activation of multiple signal cascades such as the Ras/MAPK pathway (Twal *et al*, 2001; Jin *et al*, 2006, 2007). Interestingly, FBLN1 plays an important role in human papillomavirus (HPV) E6-mediated oncogenesis (Du *et al*, 2002). Fibulin 1 can interact with HPV E6 including cancer-related HPV E6s and the transforming bovine papillomavirus 1 (BPV1) E6. Significantly, FBLN1 expression specifically inhibited both BPV1 and HPV16 E6-mediated cellular transformation. Although no significant association between HPV infection and gastric cancer has been found, it might be worthwhile to investigate whether FBLN1 is involved in the interaction of *H. pylori* with gastric epithelial cells.

In summary, we identified FBLN1 as a novel candidate TSG epigenetically silenced in gastric cancer.

## Figures and Tables

**Table 1:** Clinicopathological features of *FBLN1* methylation in gastric cancer

**Characteristics**	**Methylated (*n*)**	**Unmethylated (*n*)**	***P*-value**
*Age (years)*			
Mean±s.d.	65.4±13.4	67.5±10.5	0.486
			
*Gender*			
M	50	6	0.128
F	36	10	
			
*Helicobacter pylori infection*			
Positive	30	2	0.346
Negative	26	4	
			
*Lauren*			
Diffuse	25	6	0.058
Intestinal	38	2	
			
*Differentiation*			
Poor (or no)	33	8	0.114
Moderate	24	1	
Well	6	0	
			
*TNM stage*			
I	14	1	0.246
II	16	1	
III	24	6	
IV	21	7	

FBLN1=fibulin 1.
